# Ischemic stroke as an initial manifestation of Loeys-Dietz syndrome type 3 caused by the recurrent p.Arg287Trp variant in *SMAD3*: a case report with familial context

**DOI:** 10.3389/fgene.2026.1749577

**Published:** 2026-05-26

**Authors:** Cong-Xin Chen, Yu-yun Zhou, Zhu-quan Hong, Quan-Long Hong

**Affiliations:** Department of Neurology, First Hospital of Quanzhou Affiliated to Fujian Medical University, Quanzhou, Fujian, China

**Keywords:** arterial dissection, genotype-phenotype correlation, ischemic stroke, Loeys-Dietz syndrome type 3, Smad3

## Abstract

**Background:**

Loeys-Dietz syndrome type 3 (LDS3) is a rare autosomal dominant connective tissue disorder caused by pathogenic variants in *SMAD3.* While characteristic features include aortic aneurysm, hypertelorism, and bifid uvula, ischemic stroke as the presenting symptom has been rarely reported.

**Case presentation:**

We report a 32-year-old Chinese woman who presented with acute ischemic stroke involving the right occipital lobe and thalamus. Comprehensive vascular imaging revealed multiple arterial abnormalities, including aberrant right subclavian artery with aneurysmal dilatation, right vertebral artery aneurysmal dilatation with multisegmental stenosis, left vertebral artery multifocal segmental severe stenosis, and tortuous intracranial vessels Whole-exome sequencing identified a heterozygous pathogenic variant NM_005902.4:c.859C>T (p.Arg287Trp) in *SMAD3*. Family history was significant for aortic dissection in her father.

**Conclusion:**

This case expands the phenotypic spectrum of LDS3, emphasizing that ischemic stroke may be an underrecognized initial presentation. Early genetic screening in young stroke patients with unusual vascular features may facilitate timely diagnosis and enable appropriate preventive interventions.

## Introduction

Loeys-Dietz syndrome (LDS) is a rare autosomal dominant connective tissue disorder with an estimated prevalence of 1 in 100,000 to 1 in 150,000 individuals (van de Laar et al., 2011; Loeys and Dietz, 2008). The syndrome is characterized by a triad of arterial tortuosity, hypertelorism, and bifid uvula or cleft palate, along with widespread systemic manifestations affecting cardiovascular, skeletal, craniofacial, and integumentary systems (Meester et al., 2017). Based on the underlying genetic defect, LDS is classified into five subtypes: LDS1 (*TGFBR1*), LDS2 (*TGFBR2*), LDS3 (*SMAD3*), LDS4 *(TGFB2*), and LDS5 (*TGFB3*) (van de Laar et al., 2012). All these genes encode components of the transforming growth factor-beta (TGF-β) signaling pathway, which plays a critical role in extracellular matrix homeostasis and vascular development (Massagué, 2012). Loeys-Dietz syndrome type 3 (LDS3), caused by heterozygous pathogenic variants in *SMAD3*, was first described in 2011 (Hostetler et al., 2019). *SMAD3* encodes an intracellular mediator of TGF-β signaling that regulates gene transcription involved in cell proliferation, differentiation, and apoptosis (Massagué, 2012; Derynck and Zhang, 2003). LDS3 patients typically present with aneurysms and dissections of the aorta and its branches, early-onset osteoarthritis, skeletal abnormalities, and craniofacial features (Hostetler et al., 2019; Schepers et al., 2018). To date, more than 60 pathogenic variants in *SMAD3* have been reported worldwide, with most affecting the MH2 domain (amino acids 220–425) critical for SMAD4 heterocomplex formation and transcriptional activation (van de Laar et al., 2011; de Wagenaar et al., 2024).

Ischemic stroke in young adults has an increasing recognised genetic component, with up to 30% of cases remaining cryptogenic; monogenic connective tissue disorders, particularly those affecting the TGF-β signalling pathway, represent an underrecognised but clinically actionable aetiology (Ekker et al., 2018; Hausman-Kedem et al., 2022). Even traditional craniofacial genes like IRF6 may also contribute to perinatal arterial ischemic stroke, expanding the genetic landscape of young stroke (Perilli et al., 2025). Among these, LDS3 is rarely identified as a cause of ischemic stroke.

Here, we report a 32-year-old Chinese woman who presented with acute ischemic stroke as the initial manifestation of LDS3, caused by the recurrent pathogenic variant p.Arg287Trp in *SMAD3*.

## Materials and methods

Genetic testing: After written informed consent, whole-exome sequencing (WES) was performed using the Agilent SureSelect Human All Exon V6 capture kit on an Illumina NovaSeq 6000 platform. Mean sequencing depth was 150× with >98% of targeted regions covered at ≥20×. The *SMAD3* gene had complete coverage across all 9 exons. The candidate variant was confirmed by bidirectional Sanger sequencing. Variant interpretation followed ACMG/AMP guidelines.

## Case presentation

### Clinical presentation

A 32-year-old Chinese woman presented to our hospital with sudden-onset dizziness and blurred vision for 2 days. The symptoms began upon waking, characterized by persistent non-rotatory dizziness and bilateral visual blurring, particularly when looking to the left. The patient reported no associated symptoms, including nausea, vomiting, hearing disturbance, dysphagia, dysarthria, headache, or dyspnoea. She had no significant past medical history, including no history of hypertension, diabetes mellitus, hyperlipidemia, or smoking. She denied any history of oral contraceptive use, illicit drug use, or ergot derivative exposure. Notably, her father had a history of aortic dissection and had undergone surgical repair at another hospital. Physical examination was notable for craniofacial features including hypertelorism and mild malar flattening (Figure 1). Vital signs were within normal limits (blood pressure 128/76 mmHg, heart rate 72 beats/min). Cardiovascular, skeletal, skin, and oral examinations revealed no abnormalities; specifically, no significant joint hypermobility (Beighton score 2/9), bifid uvula, arachnodactyly, pectus deformity, skin translucency, or striae were identified—findings pertinent to the exclusion of additional LDS3 features. Neurological examination disclosed left homonymous hemianopia on confrontation testing and mild decreased sensation on the left side. The remainder of the cranial nerve, motor, cerebellar, and gait examinations were normal. NIHSS on admission was 3 (2 points for visual field defect, 1 point for sensory loss). In addition, a standardised LDS3 phenotypic assessment was performed. The complete phenotypic assessment is summarised in Table 1.

**FIGURE 1:**
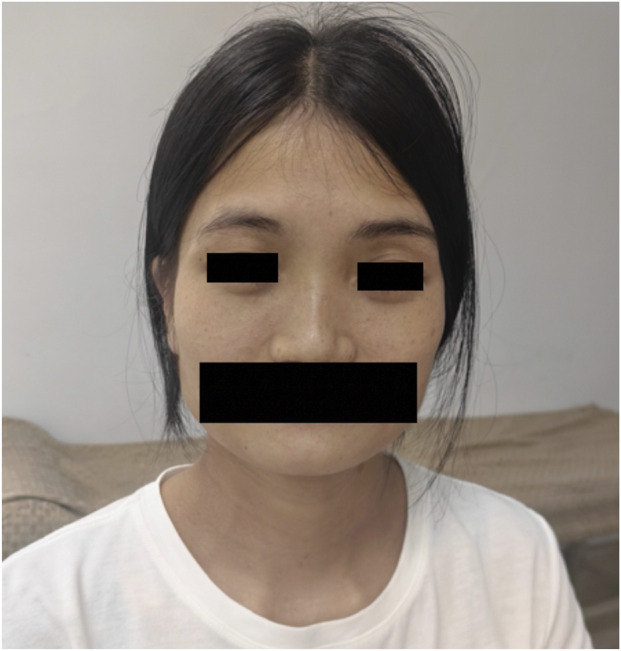
Clinical Features of the Patient. Frontal photograph showing craniofacial features including hypertelorism (increased distance between the eyes) and mild malar flattening. The patient provided written informed consent for publication of this photograph with anonymization.

**TABLE 1:** Comprehensive LDS3 phenotypic assessment of the proband.

System	Feature	Present/Absent	Details
Craniofacial	Hypertelorism	Present	Clinically evident (Figure 1)
	Malar flattening	Present	Mild
	Bifid uvula/cleft palate	Absent	Normal oral examination
	Retrognathia	Absent	Normal mandibular profile
Cardiovascular	Aortic root dilatation	Absent	Aortic root 28 mm, Z-score normal
	Arterial tortuosity	Present	Bilateral ICA and PCA
	Arterial aneurysm	Present	Aberrant right subclavian artery, max. 15 mm; right vertebral artery V1-segment aneurysmal dilatation
	Aortic dissection	Absent	Normal thoracoabdominal CTA
	Mitral valve prolapse	Absent	Normal echocardiography
Skeletal	Joint hypermobility	Absent	Beighton score 2/9
	Scoliosis	Absent	Normal spinal alignment
	Pectus deformity	Absent	Normal chest wall
	Arachnodactyly	Absent	Normal digit proportions
	Early-onset osteoarthritis	Absent	Hand and knee X-rays unremarkable
Ocular	Myopia	Absent	Normal visual acuity
	Retinal detachment	Absent	Fundoscopic examination normal
Skin	Striae	Absent	Normal skin examination
	Easy bruising	Absent	No history
	Translucent skin	Absent	Normal texture and thickness
	Atrophic scarring	Absent	None observed

### Laboratory investigations

A comprehensive workup for young stroke, including haematological, inflammatory, thrombophilic, autoimmune, and cardiac investigations, was unremarkable. Notably, LDL-cholesterol was 2.22 mmol/L, echocardiography showed normal cardiac structure and aortic root dimensions (28 mm), bubble contrast study was negative for patent foramen ovale, and 24-h ECG monitoring showed sinus rhythm without atrial fibrillation. Per the TOAST classification, the stroke was attributed to SMAD3-related arteriopathy rather than embolic stroke of undetermined source.

### Neuroimaging findings

Brain MRI with diffusion-weighted imaging (DWI) confirmed acute ischemic infarction in the right occipital lobe and thalamus. Susceptibility-weighted imaging (SWI) revealed co-localised microbleeds in the right occipital lobe, most likely representing haemorrhagic transformation (Figure 2). CT angiography demonstrated bilateral vertebral artery stenosis with post-stenotic dilatation and an aberrant right subclavian artery (arteria lusoria) with aneurysmal dilatation (maximum diameter 15 mm) at its origin. Three-dimensional reconstruction delineated the full aortocerebrovascular tree (Figures 3A,B). Digital subtraction angiography (DSA) confirmed the bilateral multisegmental vertebral arteriopathy, with the right side showing alternating aneurysmal dilatation and stenosis across V1–V4 and the left side showing multifocal severe stenosis at V2 (Figures 3C,D). Detailed quantitative vascular measurements are provided in Table 2. Thoracoabdominal CTA confirmed normal aortic dimensions.

**FIGURE 2:**
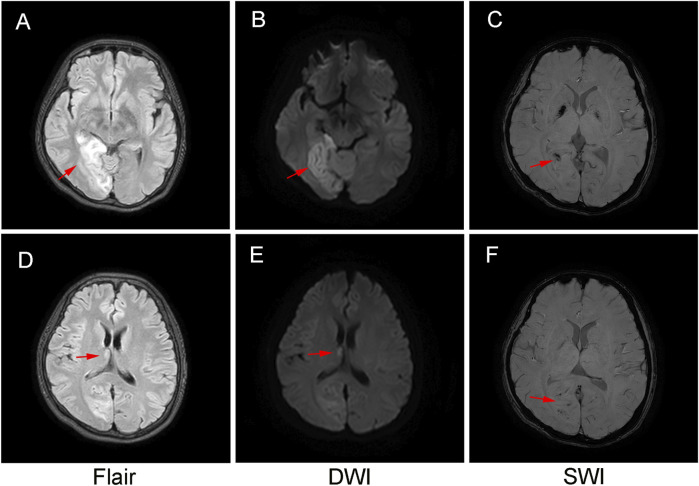
Brain Magnetic Resonance Imaging Findings. Brain MRI showing acute ischemic infarction in the right occipital lobe and thalamus. **(A,D)** FLAIR sequences demonstrate hyperintense signals (red arrows). **(B,E)** DWI sequences confirm acute infarction with restricted diffusion (red arrows) in the right posterior cerebral artery territory. **(C,F)** SWI sequences reveal multiple co-localised microbleeds (red arrows) in the right occipital lobe, most likely representing haemorrhagic transformation of acute ischemic infarction (see “Differential Diagnosis of Microbleeds” in Discussion for full differential). (FLAIR, fluid-attenuated inversion recovery; DWI, diffusion-weighted imaging; SWI, susceptibility-weighted imaging).

**FIGURE 3:**
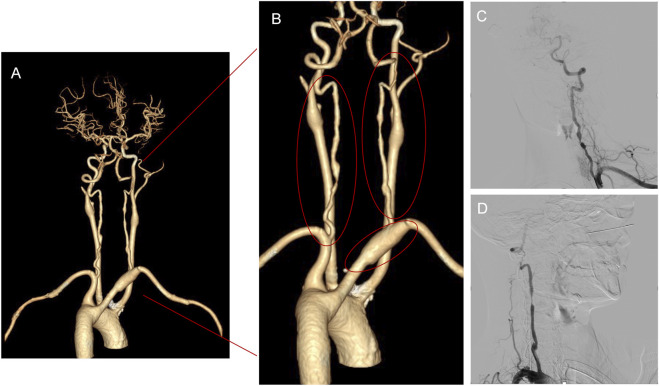
Cerebrovascular and Aortic Imaging Demonstrating Arteriopathy and Aberrant Vascular Anatomy. **(A)** Three-dimensional reconstruction of CT angiography showing the entire aortocerebrovascular tree, demonstrating aberrant right subclavian artery (artery lusoria) originating distal to the left subclavian artery with aneurysmal dilatation at its origin, and bilateral multisegmental vertebral artery abnormalities. **(B)** Magnified view of the vertebral arteries highlighting (red circles): the right vertebral artery demonstrates aneurysmal dilatation at V1, severe stenosis at V2, and moderate stenosis at V3–V4; the left vertebral artery shows multifocal segmental severe stenosis at V2 with mild post-stenotic dilatation. Quantitative measurements: see Table 2 (NASCET method for extracranial segments, WASID method for intracranial V4 segment). **(C)** Digital subtraction angiography (DSA) of the right vertebral artery in lateral view, demonstrating the multisegmental arteriopathy pattern with V2 severe stenosis (arrow) and V3–V4 moderate stenosis, with reduced distal flow. **(D)** DSA of the left vertebral artery in anteroposterior view, demonstrating multifocal segmental severe stenosis at V2 with post-stenotic dilatation and tortuous distal branches. The bilateral multisegmental vertebral arteriopathy pattern—characterised by alternating aneurysmal dilatation and stenosis—is characteristic of connective tissue disorders and represents the most likely source of posterior circulation stroke in this patient with LDS3 (see “Stroke Mechanism Analysis” in Discussion).

**TABLE 2:** Quantitative vascular phenotype.

Vessel	Finding	Measurement	Method
Right vertebral artery (V1)	Aneurysmal dilatation	Diameter 6.8 mm (normal reference ∼3.0–4.0 mm)	CTA
Right vertebral artery (V2)	Severe stenosis	∼78% luminal narrowing	NASCET, CTA/DSA
Right vertebral artery (V3–V4)	Moderate stenosis	∼52% luminal narrowing (V3, NASCET); ∼48% luminal narrowing (V4, WASID)	NASCET (V3)/WASID (V4), CTA/DSA
Left vertebral artery (V2)	Multifocal segmental severe stenosis	∼75% luminal narrowing at most stenotic point	NASCET, CTA/DSA
Left vertebral artery (post-stenotic)	Mild dilatation	Diameter 4.5 mm	CTA
Aberrant right subclavian artery	Congenital vascular variant with aneurysmal dilatation at origin (not meeting Kommerell diverticulum threshold)	Maximum diameter 15 mm at origin (note: Kommerell diverticulum defined as ≥ 30 mm in adults; current size does not meet threshold)	CTA
Bilateral ICA	Increased tortuosity	Tortuous course bilaterally	CTA/DSA
Bilateral PCA	Increased tortuosity	Tortuous course bilaterally	CTA/DSA
Thoracic aorta (aortic root)	Normal	28 mm; Z-score within normal limits	Echo/CTA
Thoracic aorta (descending)	Normal	No dilatation	CTA

CTA, computed tomography angiography; DSA, digital subtraction angiography; Echo, echocardiography; ICA, internal carotid artery; PCA, posterior cerebral artery; WASID, Warfarin-Aspirin Symptomatic Intracranial Disease method.; NASCET, North American Symptomatic Carotid Endarterectomy Trial. Stenosis percentages were measured using different validated methods according to vessel segment: the NASCET, method was applied to extracranial segments (V1–V3), in which the distal normal vessel diameter serves as the reference; the WASID, method was applied to the intracranial V4 segment, in which the proximal normal vessel diameter serves as the reference (We acknowledge that in multisegmental disease with both aneurysmal and stenotic segments, the choice of reference vessel may introduce measurement uncertainty).

### Genetic analysis

Given the young age at stroke onset, unusual vascular features, suggestive craniofacial features, and a positive family history of aortic dissection, a hereditary connective tissue disorder was suspected. WES identified a heterozygous pathogenic *SMAD3* variant (NM_005902.4:c.859C>T; p.Arg287Trp) (Figure 4), confirmed by Sanger sequencing and previously classified as pathogenic in ClinVar (VCV000043097.6). The detailed ACMG evidence assessment is provided in Supplementary Table S1 (appended at the end of the manuscript).

**FIGURE 4:**
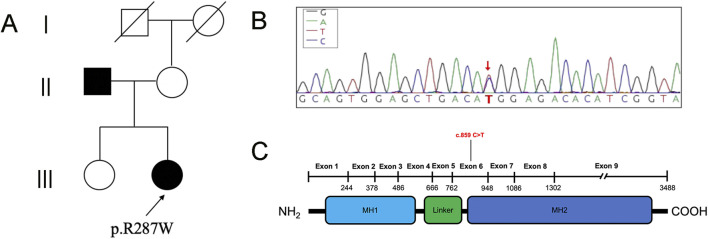
Genetic Analysis and *SMAD3* Structure **(A)** Family pedigree showing autosomal dominant inheritance. The proband (III-2, indicated by arrow) is a 32-year-old female who presented with ischemic stroke. Her father (II-1) had aortic dissection requiring surgical repair, suggesting he is a carrier of the same *SMAD3* variant (genetic testing not performed); see Discussion for explanation. **(B)** Sanger sequencing chromatogram confirming the heterozygous c.859C>T variant (red arrow) in exon 6 of *SMAD3*. The wild-type nucleotide C is partially replaced by T, resulting in the missense mutation. **(C)** Schematic representation of *SMAD3* gene and protein structure. (Top) The *SMAD3* gene comprises 9 exons with nucleotide positions indicated. The c.859C>T variant is located in exon 6, and exon 6 spans nucleotides 763–948 of the mRNA and encodes amino acids 229–290. (Bottom) The 425-amino acid SMAD3 protein contains three functional domains: MH1 domain responsible for DNA binding, linker region containing phosphorylation sites, and MH2 domain mediating SMAD4 heterocomplex formation. The pathogenic variant p.Arg287Trp (red star) is located within the MH2 domain, disrupting SMAD3-SMAD4 interaction essential for TGF-β signaling.

### Diagnosis and management

Based on clinical, radiological, and genetic findings, the patient was diagnosed with: Acute ischemic stroke (right occipital lobe and thalamus); Loeys-Dietz syndrome type 3. The patient was treated with dual antiplatelet therapy (aspirin 100 mg and clopidogrel 75 mg daily), atorvastatin 20 mg daily, and supportive care.

The antithrombotic strategy was selected following multidisciplinary team discussion, balancing ischemic recurrence risk against haemorrhagic risk:

1. Rationale for dual antiplatelet therapy (DAPT): Given the most likely stroke mechanism of artery-to-artery embolism secondary to severe bilateral multisegmental vertebral arteriopathy (see “Stroke Mechanism Analysis” in Discussion), antiplatelet therapy was deemed the most appropriate strategy. DAPT was maintained for 21 days, consistent with the CHANCE trial protocol for acute minor stroke (NIHSS ≤3). Transition to single antiplatelet therapy (clopidogrel 75 mg daily) was made on day 21. The POINT trial was referenced for comparative context but employs a 90-day DAPT duration; we selected the shorter regimen given the patient’s low NIHSS and the presence of SWI microbleeds, which were weighed against prolonged dual antiplatelet exposure (Wang et al., 2013; Johnston et al., 2018).
2. Consideration of microbleeds and arterial fragility: The SWI microbleeds observed in the right occipital lobe were co-localised with the acute infarct territory, most likely representing haemorrhagic transformation rather than widespread cerebral amyloid angiopathy (see “Differential Diagnosis of Microbleeds” in Discussion). Although LDS3-associated arterial wall fragility theoretically increases haemorrhagic risk, the limited number of microbleeds and their peri-infarct distribution were considered acceptable for short-term DAPT.
3. Rationale against anticoagulation: Anticoagulation was not selected because: (a) there was no evidence of cardioembolism (normal echocardiography, negative bubble contrast study, no atrial fibrillation); (b) no definitive evidence of arterial dissection was identified on CTA and DSA (no intimal flap, double lumen, or mural hematoma); and (c) arterial wall fragility in LDS3 may increase haemorrhagic risk with anticoagulation.
4. Long-term strategy: The patient was transitioned to clopidogrel 75 mg daily monotherapy after 21 days. The optimal antithrombotic regimen in connective tissue disorder-associated stroke remains an unresolved question that merits investigation in larger cohorts.

She was counseled regarding lifelong cardiovascular surveillance, including annual echocardiography and vascular imaging. Blood pressure control with angiotensin receptor blockers was recommended. The patient was discharged after 2 weeks with improved symptoms. NIHSS at discharge (day 14) was 1 (mild residual left homonymous hemianopia). Modified Rankin Scale (mRS) at discharge was 1 (no significant disability despite symptoms). Barthel Index at discharge was 100 (independent in all activities of daily living). At 3-month follow-up, she remained neurologically stable with no recurrent events. mRS at 3-month follow-up was 0. Formal automated perimetry (Humphrey visual field 24–2) at 3-month follow-up demonstrated complete resolution of the left homonymous hemianopia.

## Discussion

This case illustrates three critical aspects of LDS3: (1) ischemic stroke can be the initial presenting manifestation, expanding the known phenotypic spectrum; (2) comprehensive vascular imaging and genetic testing are essential in young stroke patients with atypical features; and (3) family history plays a crucial role in guiding diagnostic evaluation.

While aortic aneurysm and dissection are well-established features of LDS3, cerebrovascular events as the initial presentation remain underrecognised; stroke was not the presenting feature in the original 52-patient SMAD3 patients (van de Laar et al., 2011), and approximately 9%–16% of LDS patients have cerebrovascular abnormalities, though stroke as a first symptom remains exceedingly rare (MacCarrick et al., 2014).

### Stroke mechanism analysis

Understanding the stroke mechanism in this patient is critical for guiding secondary prevention. We systematically evaluated potential aetiologies using the TOAST classification framework: (1) Large-artery atherosclerosis: Considered unlikely given the patient’s young age (32 years), absence of traditional vascular risk factors, normal LDL-cholesterol (2.22 mmol/L), and the atypical bilateral multisegmental vertebral artery stenosis pattern (right V2 severe stenosis with V1 aneurysmal dilatation and V3–V4 moderate stenosis; left V2 multifocal segmental severe stenosis), which differs from atherosclerotic disease that preferentially affects vertebral artery origins. (2) Arterial dissection: CTA and DSA did not demonstrate classic imaging features of dissection (intimal flap, double lumen, or mural haematoma). However, subtle dissection cannot be entirely excluded, particularly given the inherent fragility of the arterial wall in LDS3. High-resolution vessel wall MRI was not performed, which is noted as a limitation. (3) Artery-to-artery embolism and haemodynamic compromise: This is considered the most probable mechanism. The right vertebral artery showed multisegmental involvement with V1 aneurysmal dilatation, V2 severe stenosis, and V3–V4 moderate stenosis; the left vertebral artery demonstrated multifocal segmental severe stenosis at V2 (see Table 2 for detailed measurements). This bilateral multisegmental arteriopathy creates conditions for disturbed flow, *in-situ* thrombosis, and artery-to-artery embolism into the right posterior cerebral artery (PCA) territory. In particular, the alternating pattern of aneurysmal dilatation and stenosis in the right vertebral artery promotes turbulent flow and stagnation zones that favour thrombus formation. The infarct distribution (right occipital lobe and thalamus) corresponds precisely to the right PCA territory, consistent with this mechanism (Caplan et al., 2006). (4) Cardioembolism: Excluded by normal transthoracic echocardiography, negative bubble contrast echocardiography (no patent foramen ovale or intracardiac shunt), sinus rhythm on 24-h ambulatory monitoring, and absence of structural cardiac abnormalities. (5) Small vessel disease: Unlikely as the primary mechanism given the territorial infarct distribution, though the microbleeds may suggest concurrent microvascular involvement (see below). In summary, bilateral vertebral arteriopathy with severe stenosis represents the most likely source of posterior circulation stroke in this patient, with haemodynamic compromise and artery-to-artery embolism as the predominant mechanism. The structural weakness of the arterial wall due to *SMAD3* dysfunction likely serves as the pathological substrate for the observed arteriopathy. The SWI microbleeds co-localised with the infarct territory most likely represent haemorrhagic transformation; cerebral amyloid angiopathy and hypertensive microangiopathy are unlikely at this age (Greenberg et al., 2009). A potential contribution of TGF-β pathway dysfunction to cerebral microangiopathy in LDS3 merits investigation in future studies (Wyss-Coray et al., 1997; Lebrin et al., 2005).

### Molecular basis and phenotypic variability of the p.Arg287Trp variant

Aberrant right subclavian artery with aneurysmal dilatation (maximum diameter 15 mm) indicates widespread vascular involvement characteristic of LDS3 (Dekker et al., 2022). This case underscores that hereditary connective tissue disorders should be considered in young stroke patients with unusual vascular features and craniofacial dysmorphism (Meester et al., 2017). Recent evidence further supports that arterial tortuosity is a clinically actionable predictor of long-term neurovascular risk in LDS, reinforcing the importance of serial vascular imaging with attention to tortuosity indices in surveillance protocols (Table 3) (Lee et al., 2025).

**TABLE 3:** Clinical Outcomes and proposed surveillance plan.

Time point	Assessment	Outcome
Admission (Day 0)	NIHSS; brain MRI/MRA; CTA; DSA; echocardiography; full laboratory workup	NIHSS 3; right PCA territory infarction; bilateral multisegmental vertebral arteriopathy (right V1 aneurysmal dilatation, V2 severe stenosis, V3–V4 moderate stenosis; left V2 multifocal segmental severe stenosis); *LDS3* diagnosed
Discharge (Day 14)	NIHSS; mRS; Barthel Index	NIHSS 1; mRS 1; Barthel 100
3 months	NIHSS; mRS; Humphrey visual field 24–2	NIHSS 0; mRS 0; hemianopia resolved
6 months	Brain MRI/MRA; neurological evaluation	Planned
12 months	Brain MRI/MRA; echocardiography; thoracoabdominal CTA/MRA; ophthalmological examination	Planned
Annually (year 2 onward)	Echocardiography; thoracoabdominal CTA/MRA; neurological evaluation; vascular tortuosity assessment (Jondeau et al., 2016)	Planned
Every 1–2 years	Skeletal survey (osteoarthritis screening); ophthalmological examination	Planned
As needed	Genetic counselling; cascade family screening	Father’s testing ongoing

The p.Arg287Trp variant identified in our patient is a recurrent pathogenic mutation in *SMAD3*, previously reported in multiple unrelated LDS3 families (van de Laar et al., 2011; Schepers et al., 2018). Located in the MH2 domain (amino acids 220–425) responsible for SMAD4 heterocomplex formation and transcriptional activation, this variant impairs SMAD3-SMAD4 interaction and reduces expression of smooth muscle contractile proteins and extracellular matrix components (Shi and Massagué, 2003). Functional characterisation has demonstrated that this variant decreases fibrillin-1 deposition, disrupts SMAD3-SMAD4 complex stability, and compromises vascular smooth muscle cell differentiation, resulting in structural weakness of the arterial wall (de Wagenaar et al., 2024). These molecular defects predispose to aneurysm formation, dissection, and accelerated atherosclerosis (Gallo et al., 2014; Cook et al., 2015).

Importantly, p.Arg287Trp demonstrates significant phenotypic variability even within families (de Wagenaar et al., 2024). While some carriers present primarily with skeletal manifestations, others develop severe vascular complications. This intrafamilial heterogeneity suggests the influence of genetic modifiers, environmental factors, or stochastic events in determining disease expression (Arnaud et al., 2021).

### Clinical management and cascade genetic screening

The diagnosis of LDS3 in a young stroke patient has profound management implications distinct from conventional stroke care. LDS3 patients require comprehensive vascular surveillance with serial imaging of the entire arterial tree to detect progressive aneurysmal changes and guide timing of prophylactic surgical intervention, along with aggressive blood pressure control (target <120/80 mmHg) using beta-blockers or angiotensin receptor blockers, with losartan showing promise in reducing aortic growth in related TGF-β pathway disorders. Patients also need lifestyle modifications including avoidance of contact sports, heavy lifting, and isometric exercises that increase dissection risk. Notably, LDS3 patients have lower surgical thresholds for prophylactic aortic repair at 4.0–4.5 cm compared to 5.0–5.5 cm in the general population. Additionally, multidisciplinary care coordination among neurologists, cardiologists, vascular surgeons, and geneticists is essential for comprehensive management of these patients (MacCarrick et al., 2014; Lacro et al., 2014; Isselbacher et al., 2022; Hiratzka et al., 2010; Groenink et al., 2013; Habashi et al., 2006; Majersik et al., 2021; Braverman et al., 2015; Boodhwani et al., 2014). Serial assessment of vascular tortuosity has accordingly been incorporated into the long-term surveillance protocol (Lee et al., 2025) (Table 3).

The patient’s father had aortic dissection requiring surgical intervention at age 58, strongly suggesting he carries the same *SMAD3* variant. Unfortunately, genetic testing was not performed due to technical limitations. The genotype-phenotype correlation discussed herein is therefore established primarily through the proband’s clinical and genetic data combined with published literature on the p.Arg287Trp variant, rather than direct familial co-segregation. The absence of segregation testing weakens the direct familial genotype-phenotype claim; however, the father’s aortic dissection phenotype—a hallmark of LDS3 — provides strong indirect evidence of shared genetic aetiology. Cascade genetic testing of at-risk family members is crucial for identifying asymptomatic carriers who require surveillance imaging, facilitating informed reproductive decisions, and enabling implementation of preventive strategies that may avert life-threatening complications (Jondeau et al., 2016; Verhagen et al., 2018; Gollob et al., 2011). Current guidelines recommend that first-degree relatives of LDS3 patients undergo genetic testing regardless of clinical features (MacCarrick et al., 2014; Boodhwani et al., 2014).

We propose that genetic testing for hereditary connective tissue disorders, including LDS3, should be considered in young adults (<50 years) with ischemic stroke when one or more of the following features are present: multiple arterial aneurysms, dissections, or marked tortuosity; positive family history of aortic disease or sudden cardiac death; craniofacial dysmorphism (hypertelorism, malar hypoplasia, bifid uvula); skeletal features (scoliosis, joint hypermobility, early-onset osteoarthritis); or absence of traditional vascular risk factors (Meester et al., 2017; Verhagen et al., 2018). Early genetic diagnosis enables cascade screening and implementation of preventive strategies (Gollob et al., 2011).

### Limitations

Limitations of this study include the absence of segregation testing in the father, a 3-month follow-up period, lack of high-resolution vessel wall MRI to definitively exclude subtle arterial dissection, and the absence of patient-specific functional studies. Future research should focus on genetic modifiers, risk-stratification biomarkers, and prospective therapeutic trials (Arnaud et al., 2021; Pinard et al., 2019).

## Conclusion

This case expands the phenotypic spectrum of LDS3 by demonstrating ischemic stroke as an initial manifestation in a young adult with the recurrent p.Arg287Trp variant in *SMAD3*. It underscores the importance of considering hereditary connective tissue disorders in young stroke patients with unusual vascular features, suggestive craniofacial characteristics, or family history of aortic disease. Early genetic diagnosis enables cascade screening and implementation of surveillance protocols that may prevent life-threatening cardiovascular complications.

## Data Availability

The original contributions presented in the study are included in the article/Supplementary Material, further inquiries can be directed to the corresponding author.
