# Supplementary material for: Ischemic stroke as an initial manifestation of Loeys-Dietz syndrome type 3 caused by the recurrent p.Arg287Trp variant in SMAD3: a case report with familial context
Source: Front Genet. 2026 May 26;17:1749577. doi: 10.3389/fgene.2026.1749577 (PMC13245937; doi:10.3389/fgene.2026.1749577)
Supplement: Supplementary file 1 [file Table1.docx]

**Supplementary Table S1. ACMG/AMP evidence summary for *SMAD3* NM_005902.4:c.859C>T (p.Arg287Trp)**

| ACMG criterion | Strength | Evidence (case-specific) | Key references / databases |
| --- | --- | --- | --- |
| PS1 | Strong | Same amino-acid change as previously established pathogenic variant (p.Arg287Trp) reported in multiple LDS3 families. | ClinVar VCV000043097.6; published LDS3 cohorts [1,8] |
| PS3* | Moderate | Functional studies reported damaging effect of p.Arg287Trp on SMAD3 function (e.g., impaired SMAD3–SMAD4 complex formation; reduced TGF-β–responsive signaling). | de Wagenaar et al., 2024 [9] |
| PM1 | Moderate | Located in MH2 domain (aa 220–425), a critical functional region and mutational hotspot for SMAD3. | Domain knowledge; LDS3 variant distribution [1,9] |
| PM2 | Moderate | Absent/extremely rare in population databases (gnomAD). | gnomAD; ClinVar |
| PP2 | Supporting | Missense variant in a gene with low rate of benign missense variation and where missense is a common mechanism of disease. | Gene-level constraint; ACMG framework |
| PP3 | Supporting | Multiple computational tools predict deleterious effect (e.g., REVEL 0.871; CADD 29.5; SIFT damaging; PolyPhen-2 probably damaging). | In silico tools as reported in this study |

** PS3 applied at Moderate strength as functional evidence derives from published literature on the identical variant rather than independent patient-derived assays (per ClinGen PS3/BS3 Calibration recommendations).*
